# *Melissa officinalis* extract improved high-fat-diet-induced anxiety-like behaviors, depression, and memory impairment by regulation of serum BDNF levels in rats

**DOI:** 10.22038/AJP.2024.24343

**Published:** 2024

**Authors:** Kazem Hatami, Majid Hassanpourezatti, Mohsen Khalili

**Affiliations:** 1 *Department of Biology, Basic Sciences School, Shahed University, Tehran, Iran*; 2 *Department of Physiology, School of Medicine, Shahed University, Tehran, Iran*

**Keywords:** Obesity, Mellissa officinalis, BDNF, Rats

## Abstract

**Objective:**

*Melissa officinalis* (MO) hydroalcoholic extract has shown neuroprotective effects. We assess the possible therapeutic effects of *Melissa officinalis* extract (MOE) on blood biochemical and Brain-Derived Neurotrophic Factor (BDNF) levels as well as neurobehavioral consequences of high-fat-diet (HFD)-induced obese rats.

**Materials and Methods:**

Eighty male Wistar rats weighing between 180 and 220 g were divided into two groups at the beginning of the experiment and fed with normal diet (ND) or HFD for 5 weeks. Then, each group was divided into four subgroups (10 rats in each group) and treated daily with MOE (50, 100, 150 mg/kg, intraperitoneal) or vehicle for another two weeks. At the end of the experiments, fasting blood glucose (FBG), blood lipid profile, and serum brain-derived neurotrophic factor (BDNF) levels were measured. The sucrose preference test (anhedonia and depression), open field test (locomotor), elevated plus maze (anxiety), Y-maze (working memory), and Morris water maze test (spatial memory) were done.

**Results:**

Feeding with HFD for 7 weeks caused obesity, anhedonia, anxiety, depression and learning and memory disorders in rats and a decrease in serum BDNF level. Administration of MOE at 100 or 150 mg/kg to HFD-fed rats decreased weight gain, FBG, and serum levels of total low-density lipoprotein cholesterol and increased serum BDNF levels. It also improved changes in locomotor activity, anxiety, depression, and learning and memory in HFD-fed rats.

**Conclusion:**

The results show that MOE has a therapeutic effect on model rats with HFD-induced metabolic and neurobehavioral abnormalities through regulation of BDNF secretion.

## Introduction

Obesity and related neurobehavioral complications show an increasing trend in the world population (Grover et al., 2023; Farruggia and Small, 2019). Several studies showed that high-fat diet (HFD) consumption is associated with behavioral abnormalities (Wang et al., 2023c). It is documented that even short-duration HFD consumption leads to obesity, hyperglycemia, and hyperlipidemia, and is followed by anhedonia, depression, and anxiety, as well as impairment in spatial memory in rodents (Volcko et al., 2020; Dutheil et al., 2016; Lof et al., 2022). HFD consumption is a relevant model for studying human obesity-related metabolic complications and obesity-related behavior (Noronha et al., 2019; Labban et al., 2020). It is suggested that decrease in the blood brain-derived neurotrophic factor (BDNF) levels that occurs after HFD consumption is related to insulin resistance and impairment of synaptic plasticity (Karczewska-Kupczewska, et al., 2012; Liu et al., 2015). Indeed, BDNF is abundantly expressed in the central and peripheral nervous system, crossing the blood-brain barrier in both directions, and affecting synaptic plasticity (Pan et al., 1998).

 The alteration of serum BDNF levels significantly correlates with obesity-related disorders, including anxiety, depression, and neurodegenerative diseases (Chan et al., 2021; Arévalo and Deogracias, 2023). It was established that a reduction in serum BDNF level leads to cognitive impairment in human and rodent models of obesity (Sun et al., 2018a; Naha et al., 2018; Katuri et al., 2021; Hoseindoost et al., 2019). It is acknowledged that BDNF plays a critical role in neural cue reactivity to food stimuli and food craving in obese persons (Bumb et al., 2021). Earlier studies have pointed out that circulatory BDNF stimulates insulin production, represses glucagon production in the pancreas, and regulates cholesterol homeostasis and lipid metabolism (Rozanska et al., 2020; Bathina and Das, 2015; Suzuki et al., 2007). 

 It has been reported HFD influenced synaptic plasticity and cognition by changing the amount of BDNF production (Wu et al., 2004). BDNF administration is suggested as a rescue treatment for memory deficits (Braschi et al., 2021). Therefore, serum BDNF seemed to be a putative target for plant extracts and implicated in the amelioration of metabolic deregulations related to obesity.

The *ethanolic* *extract* *of* Melissa officinalis possesses many pharmacological and biological effects, such as anti-oxidative, anti-inflammatory, and anti-diabetic activities (Draginic et al., 2022). The positive effects of MO on visceral obesity, insulin resistance, hypercholesterolemia, and central nervous system (CNS) disorders have also been reported (Lee, 2020; Noguchi-Shinohara et al., 2020; Naseri et al., 2021; Zarei et al., 2014; Zam et al., 2022). Some researchers have pointed to beneficial effects of some active compounds found in MO on cognitive processes through BDNF modulation (Neshatdoust et al., 2016). Nevertheless, the effect of MO treatment on HFD-related biochemical and neurobehavioral dysfunction as well as serum BDNF in rats, has not been studied already. MO aqueous extract contains variety of phenolic compounds: protocatechuic, caftaric, caffeic, ferulic, and cichoric acids and flavonoid luteolin which have shown anti-hyperlipidemic potential (Ramanauskiene et al., 2016; Aqeel et al., 2018). In addition, a previous study showed that supplementation of this plant extract along with various lipid-lowering drugs can be effective in improving LDL levels in patients with hyperlipidemia (Jandaghi et al., 2016).

 Therefore, identification of plant extracts that can modulate biochemical and neurobehavioral disorders in HFD-induced obesity will facilitate the way forward for the development of highly efficient drugs to treat obesity-related complications. According to the beneficial effects mentioned above, we aimed to examine the consequences of *Melissa officinalis *extract treatment on HFD-induced neurobehavioral alteration and serum BDNF levels in rats. 

## Materials and Methods

### Animals

Eighty male Albino Wistar rats (age 9-10 weeks) with weights in the range of 180-220 g were used in the present study. Animals were housed three per cage in polypropylene cages with a relative humidity of 45–55%, at 22±2°C and a 12 hr light/dark cycle, and they had free access to food and water. All procedures followed the instruction for the care and use of laboratory animals provided by the Shahed University Ethics Committee (No. 2019-2-2-I-5623). 

### Plant material

*Melissa officinalis* L. (Lamiaceae) leaves (voucher No. 408) were purchased from a traditional herbal shop in Kermanshah, Iran. The plant was identified in the Department of Biology, Faculty of Basic Sciences School of Shahed University. 

### Preparation of hydroalcoholic extract

Ethanol extract was obtained by the maceration method (Soulimani et al., 1991). Briefly, 30 g of cryo-ground powder was added to 300 ml of 30% ethanol (w/w) and was left to macerate at 22°C for 12 hr; and then macerated at 35°C for 12 hr and the resulting extract was filtrated, after which the solvent was evaporated under 35°C and freeze-dried. The final product yielded 18 mg for 100 mg of dry plant.

### Experimental design

At First, animals were divided into two groups: normal diet (ND) group received a normal diet (10.6% fat). The HFD group received fatty diet contained standard diet (59.6%) with lard (40% fat) for 5 consecutive weeks (Al-Thepyani et al., 2022). Normal diet was purchased from Pars Dam Company, Tehran, Iran. At the end of the 5 weeks, each group was divided into four subgroups (n=10) and daily treated intraperitoneally with vehicle or MOE (50, 100, and 150 mg/kg, i.p.) for two weeks (Ghazizadeh et al., 2020). The body weight of rats was measured at the start and the end of the study.

### Sampling and assessments

**Figure 1:**
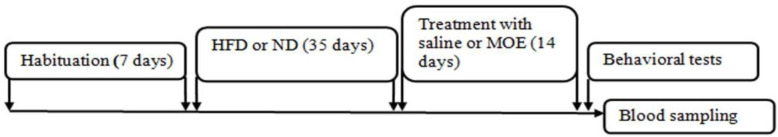
A schematic diagram of the experimental timeline. After 5 weeks, the matched normal diet (ND) and high-fat diet rats (HFD) were divided into the following groups: ND+VEH, ND+MOE (50, 100, and 150 mg/kg/day), and HFD +VEH, HFD+MOE (50, 100, and 150 mg/kg/day). All agents injections were performed daily intraperitoneally between 12:00-1:00 PM for two weeks. The rationale for injection dosage and route was based on previously published reports describing the neuroprotective effects of this intervention.

At the end of behavioral studies, animals were anesthetized using ketamine (80 mg/kg, i.p.) and xylazine (12 mg/kg, i.p.). Blood samples were collected directly from the heart puncture and centrifuged to collect serum for BDNF assays. Triglyceride (TG), total cholesterol (TC), low-density lipoprotein cholesterol (LDL), and high-density lipoprotein cholesterol (HDL) levels were determined using enzymatic kits supplied by using the Pars Azmoon commercial kit (Tehran, Iran) based on the photometric method. Values are expressed in mg/dL. Rat BDNF ELISA kit was purchased from ZellBio GmbH (Germany). A schematic diagram of the experimental timeline is shown in Figure 1. 

### Sucrose preference test (SPT)

Anhedonia was evaluated using the sucrose preference test (SPT). After 24 hr of water deprivation, rats were allowed to drink freely from bottles filled with sucrose solution (1%) and water. Water and sucrose solution consumptions were measured within one hour (Aryanezhad et al., 2021). SPT was calculated as sucrose intake / (sucrose intake + water intake) × 100%

### Elevated plus maze (EPM)

This test investigated anxiety-like behavior (Salmani et al., 2022). The EPM apparatus was purchased from Borj Sanat (Borj Sanat Company, Tehran, Iran). It was made of four plus-shaped arms: two open arms and two closed arms (50 x 10 x 40 cm), and made from wood. It was placed 50 cm above the ﬂoor level. The comparison between groups was based on the time spent in the open arms as a measure of anxiety and the number of entries into the closed arm as a measure of locomotor activity.

### Open field test (OFT)

The open field equipment is a 100×100×40 cm wooden box (Tajhiz Gostar Omid Iranian. Co, Karaj, Iran), and the floor of the apparatus was divided into two smaller segments called central and peripheral zones. The locomotor activity was evaluated by the total distance traveled in the central and peripheral areas, and time spent in peripheral and central areas (Kawai et al., 2007).

### Y-maze task

Y-maze allows for evaluating spatial short-term memory. The Y-maze consists of three interconnected equal (40 × 10 × 15 cm) arms with 120° angle. Rats were placed in the center of the Y-maze, and allowed to explore the maze freely for 8 min; the sequence of entering the arms and the total number of entering the arms were manually recorded. The right consecutive alternation was ABC, BCA, or CAB, when each arm in the maze was assigned as A, B, and C (Park et al., 2020a). The percentage of spontaneous alternation was defined as entering all three arms in three sequential arm entries. This value can be calculated by the following equation: 

% spontaneous alternation behavior = [(Number of right alternations)/(Total number of arm entries – 2)] × 100.

### Morris water maze (MWM) test

The MWM test assesses spatial memory (Asadbegi et al., 2017). This maze had a black circular pool (diameter 180 cm and height 60 cm) filled with water at a temperature of 22±1°C to a depth of 25 cm in the center of the room with different cues for spatial guidance on the wall. The pool was divided into four quadrants, and a black escape platform was placed 1 cm below the water surface in the center of quadrant 2. Training consisted of three trials. A video camera (Nikon, Japan) installed above the pool and linked to a tracking system, recorded the parameters. On the 4th day, each rat performed a single probe trial (90 sec), and no platform was present during the probe trials. Escape latency, distance travelled, and number of times crossing the platform were recorded in each trial.

### Statistical analysis

Assessment of groups was accomplished by two-way Analysis of variance (ANOVA) followed by Bonferroni's multiple comparisons test. All analyses were performed in GraphPad Prism 5 software. A p-value of less than 0.05 was considered statistically significant. All data are presented as Means±SEM.

## Results

### Body weight analysis

**Figure 2:**
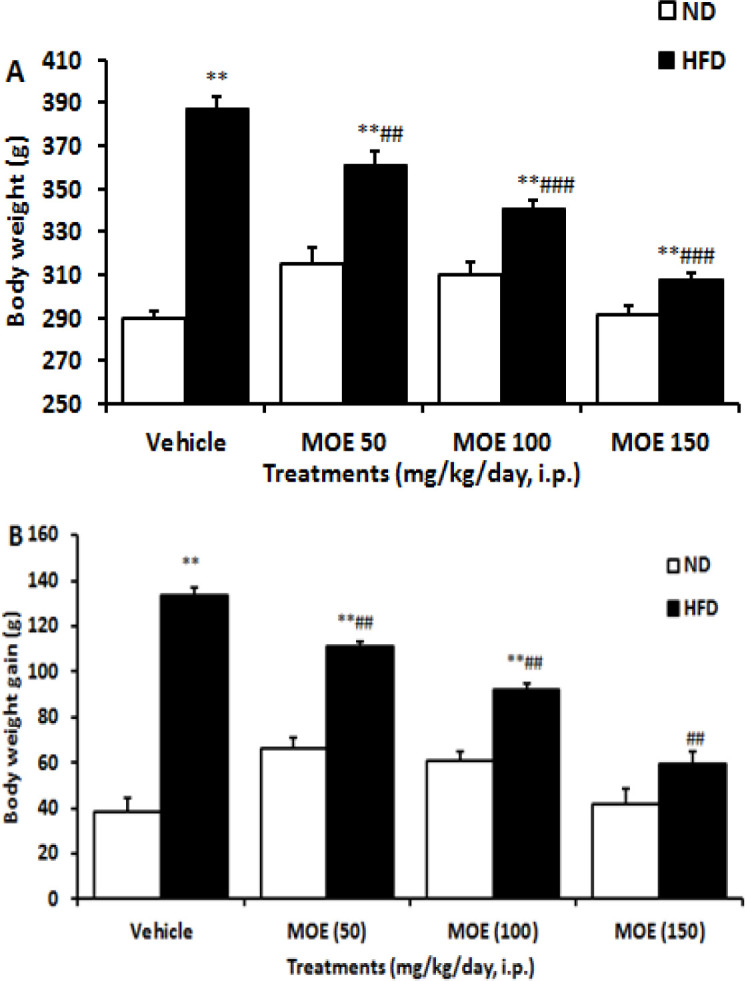
Effects of two week MOE (50, 100, and 150 mg/kg, i.p.) treatment on (A) mean body weight and (B) body weight gain of rats in normal diet (ND) and high-fat diet (HFD) fed groups (n=10 in each group). Data are presented as mean±SEM. **p<0.01 compared to the ND group. ##p<0.001 and, ###p<0.001 compared to the HFD group.

The results of the statistical tests showed that compared to the control rats, the rats fed with HFD were significantly (p<0.0001) heavier at the end of the experiments (Figure 2). In the ND group, treatment with different doses of MOE had no significant effect on the weight gain of rats compared to the vehicle-treated group. In the HFD group, MOE treatment dose-dependently suppressed the body weight gain of rats (p<0.001). 

### Biochemical determinations

**Fasting blood glucose (FBG) level**

**Figure 3:**
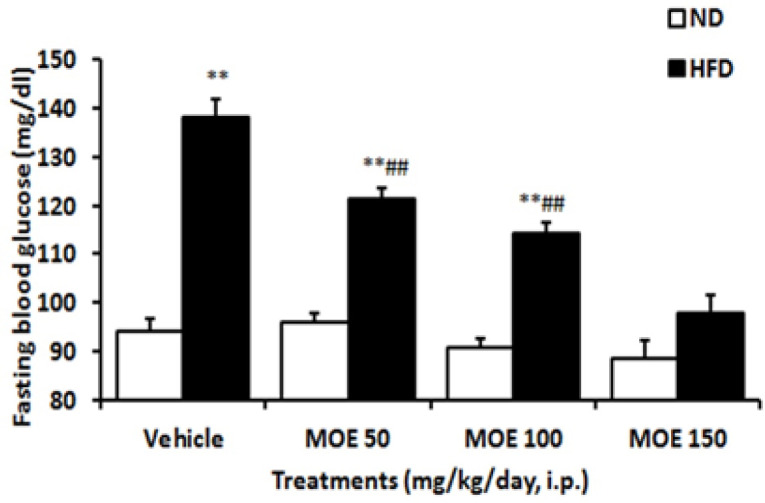
Effect of two weeks MOE treatment on fasting blood glucose (FBG) of normal diet (ND) and high-fat diet (HFD) fed rats. Data were analyzed using two-way ANOVA and post-hoc Bonferroni test. **p<0.01 compared with ND group, and ##p<0.01 compared with HFD group.

The effects of MOE treatment on FBG of ND and HFD-induced obese rats are shown in Figure 3. FBG values were significantly (p<0.001) higher in HFD than in ND group at the end of the experiments. In the ND group, daily treatment of rats with all doses of MOE for two weeks did not significantly affect their FBG levels. But administration of MOE dose-dependently (p<0.001) decreased FBG in HFD-fed rats, and even the administration of MOE (150 mg/kg/day) for two weeks was able to reduce the FBG of HFD rats to the level of ND rats.

### Serum lipid profile

As shown in Table 1, there was a significant (p<0.001) increase in serum TC, LDL and TG levels with significant (p<0.01) decreases in HDL when compared to the ND group. There was no significant difference in lipid profile values between the MOE-treated ND and HFD groups. The daily administration of MOE (100 and 150 mg/kg, i.p.) for two weeks significantly (p<0.01) decreased the TC, but only at 150 mg/kg/day significantly (p<0.01) decreased TG and LDL levels in HFD-fed rats. Additionally, HFD-fed rats treated with all doses of MOE had significantly (p<0.01) lower HDL levels compared with ND group.

### Sucrose preference test (SPT)

**Figure 4:**
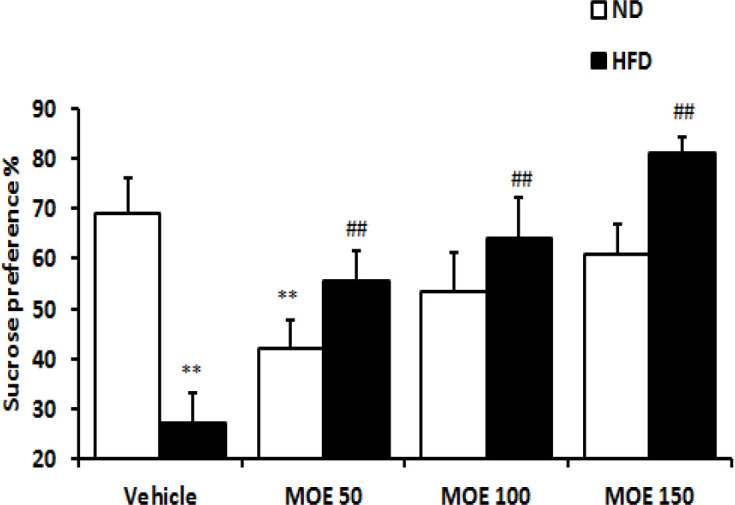
The effects of MOE on the high-fat diet (HFD)-induced anhedonia behavior in the sucrose preference test. **p<0.01 vs. vehicle-treated ND group, and ##p<0.001 vs. vehicle-treated HFD group.

As shown in Figure 4, sucrose consumption in the HFD group significantly (p<0.01) reduced when compared with the ND group. MOE-treated HFD rats showed a dose-dependent significant (p<0.01) increase in sucrose intake at the end of two weeks of treatment. Administration of MOE 50 mg/kg/day for two weeks reduced the sucrose preference in the ND compared to the vehicle-treated ND group (p<0.05). Treatment with the MOE caused a significant (p<0.001) attenuation of anhedonia behavior in HFD rats contrary to ND rats. 

### Elevated plus maze (EPM) performance

**Figure 5:**
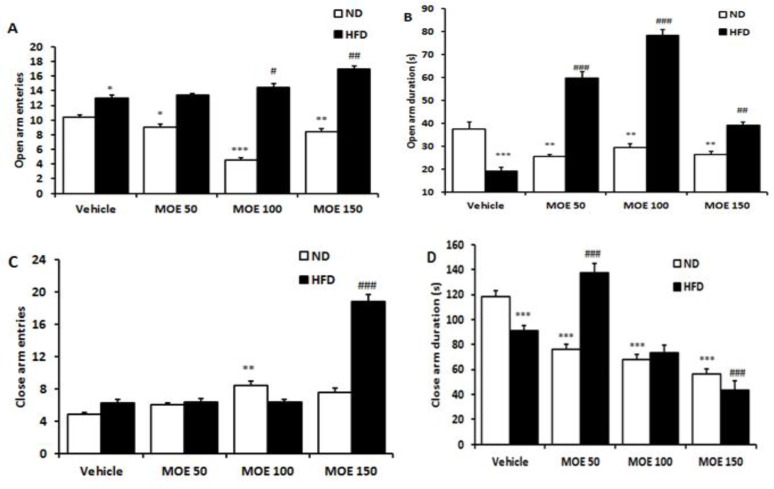
The percentage of open-arm entries (A), time spent in open arms (B), closed-arm entries (C), and time spent in closed-arm (D) of the elevated plus-maze by rats (n = 10). **p<0.01 compared with the vehicle-treated in normal diet (ND) rats and #p<0.05, ##p<0.01 and, ###p<0.001 compared with the vehicle-treated in HFD rats.

In the EPM test, HFD fed led to an increase in the open arms entries (p<0.05), but a decrease in time spent (p<0.001) and no effect on the closed arms entries and a decrease in time (p<0.001) (Figures 5 A, B, C, and D). MOE at all doses decreased the frequency of entry (p<0.001) and time spent in the open arms in the ND group (p<0.001) (Figures 5 A, B, C, and D). Administration of MOE (100 and 150 mg/kg/day) increased the frequency of open arm entries in HFD group compared to the vehicle-treated HFD group (Figures 5A and B). In addition, daily administration of 50 and 150 mg/kg of MOE in HFD group significantly (p<0.01) enhanced the time spent in the closed arm, and at 150 mg/kg, increased the number of entries into the closed arms (p<0.01) (Figures 5C and D). 

### OFT performance

**Figure 6:**
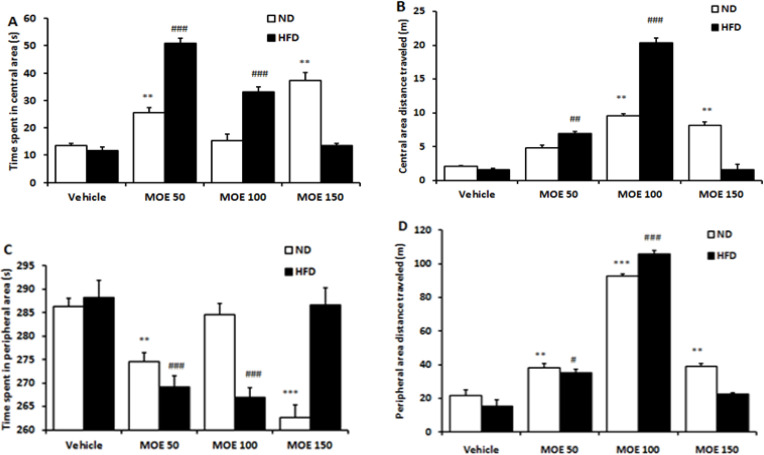
The time spent (A), and traveling distance (B) in the central area, as well as time spent (C) and traveling distance (D) in the peripheral area of the open field apparatus. *p<0.05, **p<0.01 and ***p<0.001 show differences between the ND and ND+MOE treated groups. #p<0.05, ##p<0.01, and ###p<0.001 show differences between the HFD and HFD+MOE treated groups.

In the open field, the vehicle-treated HFD rats showed no significant differences in all parameters (time spent and traveled distance in central and peripheral areas) compared to the ND rats. In comparison to the HFD group, HFD + MOE50, and HFD + MOE100 had increased (p<0.001) time spent in the central area; HFD + MOE50 and HFD + MOE100 had increased (p<0.01 and p<0.001) traveled distance in the central area; and HFD + MOE50, and HFD + MOE100 had decreased time spent in the peripheral area (p<0.01) and HFD + MOE50 and HFD + MOE100 had increased traveled distance in peripheral area (p<0.001) in the open field (Figures 6 A, B, C and D). 

### Spontaneous alternation behavior in Y-maze

**Figure 7:**
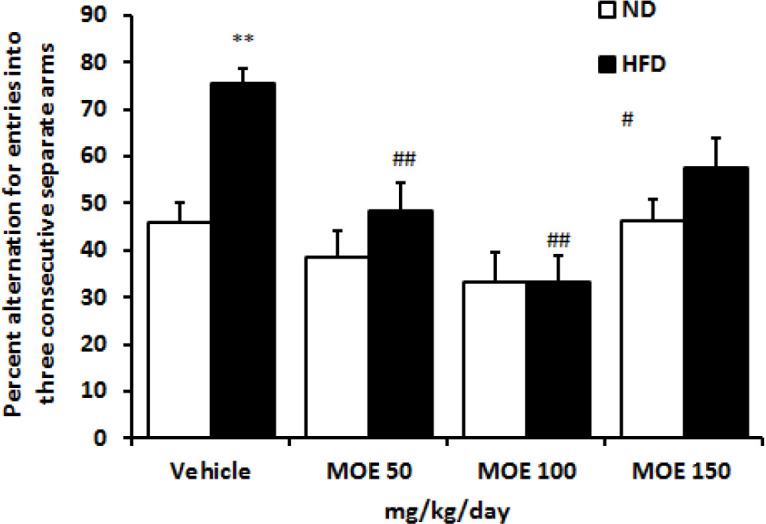
Effect of two weeks’ treatment with MOE (50, 100 and 150 mg/kg) on the percentage of spontaneous alternation (actual alterations/maximum alterations× 100) for arms entries in Y-maze for the ND and HFD groups. #p<0.05 and ##P < 0.01 MOE treated vs. vehicle-treated in HFD group. ND: normal diet, HFD: high-fat diet, MOE: *Melissa officinalis* extract.

HFD consumption increased the percentage of correct alterations in the Y-maze compared with the ND group (Figure 7). No significant changes were detected in the percentage of correct spontaneous alternation behavior percent in all MOE-treated ND-fed groups compared with vehicle-treated ND-fed rats. However, lower percentage of correct alternations was observed in MOE-treated HFD groups compared with the HFD group (p<0.01).

### Morris water maze test acquisition and retention

**Figure 8:**
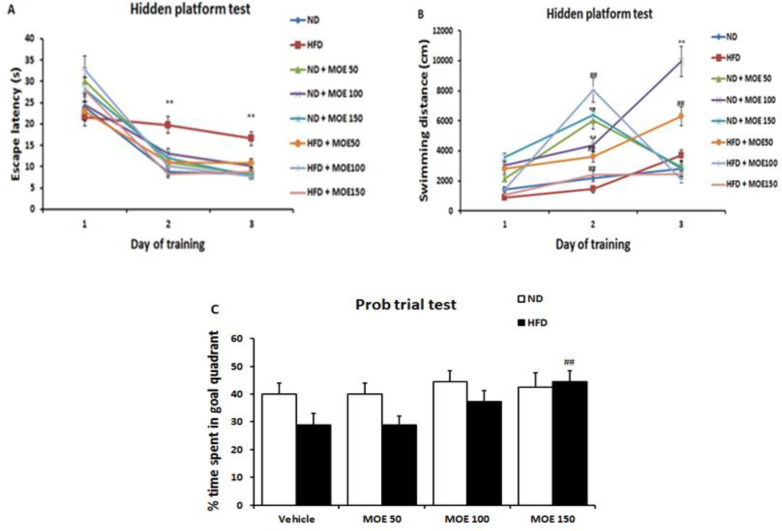
Latency times (A), swimming distance (B), % time spent in goal quadrant in Morris water maze test. Values are mean±S.E.M. *p<0.05 and **p<0.01 vs. Control group; #p<0.05, ##p<0.01 and ###p<0.001 HFD vs. HFD + MOE group. ND: normal diet, HFD: high-fat diet, MOE: *Melissa officinalis* extract.

As shown in Figures 8 A and B, the analyses of the latency to find the platform and swimming speed have significantly decreased and increased, respectively, during the three days of training in ND rats. The latency in the HFD group was longer than that of the ND groups (days 2 to 3, p<0.01). Also, the swimming speed in the HFD group was slower than that of the ND groups (day 3, p<0.01). 

The rats in MOE-treated HFD groups showed a dose-dependent decrease in latency compared with the HFD group. The HFD-fed rats spent significantly less time in the correct quadrant compared to the ND rats in the probe trial, while the group treated with the dose of 150 mg/kg of MOE for two weeks significantly (p<0.01) increased the time in the target quadrant (Figures 8 C and D), and with all the doses of MOE significantly (p<0.001) increased the number of times crosses the platform (Figures 8E). 

### Serum BDNF levels

**Figure 9:**
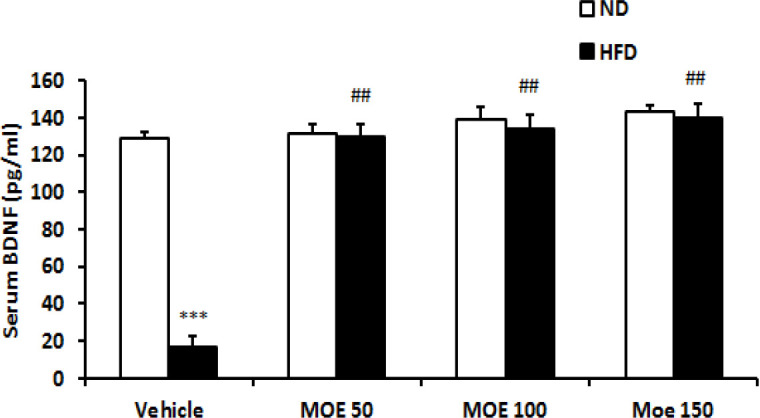
The BDNF concentration (pg/ml) in the serum of rats. *p<0.05 and **p<0.01 MOE treated vs. vehicle-treated in ND group. #p<0.05 and ##p<0.01 MOE treated vs. vehicle-treated in HFD group. ND: normal diet, HFD: high-fat diet, MOE: *Melissa officinalis* extract.

The serum concentration of BDNF in the HFD group was significantly lower than that of the ND group (p<0.05) at the end of the experiment (Figure 9). The serum BDNF was significantly increased in the HFD group after treatment with all doses of MOE compared to vehicle-treated HFD group (p<0.001). Its level remained unaffected by MOE treatment in the ND group.

## Discussion

In the present study, MOE treatment for two weeks reduced anhedonia, anxiety, and depressive-like behaviors and improved spatial learning and memory with increased serum BDNF concentration in the HFD-fed rats. Recent evidence suggests a crucial role for the effect of high blood lipid in the pathophysiology of psychiatric disorders (Schneider et al., 2017). Moreover, our current study showed that serum BDNF significantly decreased after HFD consumption, which indicates a potential role of BDNF in the pathogenesis of obesity-induced neurobehavioral and cognitive impairment. There is emerging evidence showing the potential of plants active ingredients in modulating behavioral alteration in HFD-induced obesity (Arika et al., 2019). A close relationship between lipotoxicity and anxiety, anhedonia, depression and reduction of BDNF in the brain of animals and humans has also been suggested (Seabra da Silva et al., 2022; Hidalgo-Lanussa et al., 2020; Park et al., 2011b). Increasing evidence indicates that hyperlipidemia and hypercholesteremia could lead to the down-regulation of BDNF and inhibition of hippocampal neurogenesis (Zheng et al., 2023), which ultimately causes memory and cognition impairment (Engel et al., 2019). In response to short-term HFD consumption, impairment occurs in the hippocampal- and amygdala-based cognitive function of microglial cells (Spencer et al., 2019). The current study, in accordance with previous reports, MOE can be a candidate for treating neurological complications caused by HFD. BDNF, as a signal transduction mechanism of action of MOE, has demonstrated an inverse correlation with the percentage of body fat, blood glucose, lipid profile indices, insulin sensitivity, and linking energy metabolism and cognitive function (Gyorkos et al., 2019; Kotlega et al., 2020). A close association between HFD-induced diabetes and obesity activated the transcription factor cytosine-cytosine-adenosine-adenosine-thymidine (CCAAT)/enhancer binding protein β (C/EBPβ) in hippocampal neurons, which repressed BDNF expression and caused depression-like behavior has been frequently suggested (Li et al., 2022). The study of different species has confirmed the direct relationship between the level of BDNF in brain tissue and the concentration of BDNF in the blood (Klein et al., 2011). Indeed, BDNF is a crucial transcription factor that plays a stimulatory role in regulating fat metabolism (Noble et al., 2011). Several previous investigators have found that type of diet could affect BDNF signaling in patients suffering from metabolic syndrome. On the other hand, stimulation of the BDNF signaling pathway can enhance sucrose preference, anxiolytic-like activities, and related learning and memory in a variety of depression models (Yi et al., 2014; Quesseveur et al., 2013; Wang et al., 2017b), indicating that stimulating BDNF signaling exhibited strong neurobehavioral modulating effects. A study has suggested that alteration of BDNF production is also involved in the neuropathic processes related to obesity, such as depression (Akbarian et al., 2018). Circulatory BDNF level is a molecular biomarker for anhedonia, anxiety, and depression status (Curi et al., 2021; Emon et al., 2020; Li et al., 2022). Studies have also reported that stimulation of BDNF production could improve anxiety, depression, and memory in murine (Murawska-Ciałowicz, 2021; Miranda et al., 2019). It was shown that consumption of HFD significantly decreased motor activity in the open field test and sucrose consumption (Han et al., 2021; Kurhe and Mahesh, 2017). 

Miyanishi and Nitta found that BDNF improved anhedonia and played a vital role in the dopaminergic mesolimbic circuit (Miyanishi and Nitta, 2021). Behavioral analysis of animals showed a clear increase in parameters measured in open field activity of animals after administration of 100 mg/kg. But these changes may rely on multiple factors acting simultaneously. Also, it is possible that this behavior is partly due to side effects of MOE. The application of combined microscopic and molecular technique-based approaches is suggested for future studies. It was not shown that locomotor activity increased after MOE administration, but did not specify the mechanism, nor it is clear whether these data are consistent with our study (Ozarowski et al., 2016).

We also found that MOE treatment for two weeks significantly reversed the HFD effects and returned serum BDNF level to control value. Previous reports indicated that MOE can inhibit hepatotoxicity in rats (Shahani et al., 2023). A large amount of data, *in vivo* and *in vitro*, support the role of BDNF in regulating of body weight, metabolic homeostasis, major depressive disorder, schizophrenia, and bipolar disorder (Iu and Chan, 2022). In support, another study also showed that mitigating effect of a mixture containing MOE to reverse the effect of HFD on lipid peroxidation by enhancing glutathione, catalase, and superoxide dismutase (Cho et al., 2021). Also, it was demonstrated that MOE improved obesity and insulin sensitivity in HFD-fed obese C57BL/6J Mice (Lee et al, 2020). Moreover, the anxiolytic and antidepressant-like effects of MOE in rats were demonstrated by using an elevated plus maze, forced swimming, and open field tests (Taiwo et al., 2012). In agreement with our observations, MOE supplementation had a reducing effect on body weight gain through appetite modulation. Finally, in agreement with previous research, we found that MOE increases BDNF expression (Naseri et al., 2021; Yoo et al., 2013). BDNF improves anhedonia, anxiety, and depression (Levchuk et al., 2020; Sleiman et al., 2016). Thus, the present study demonstrated a potential anti-obesity effect of MOE on the HFD-induced changes in neurobehavioral function. The administration of MOE to HFD-fed rats improved the time spent, and distance traveled in the central area in our open field tests. In this line, consumption of HFD diet was shown to induce anxiety behavior in rodents as tested in EPM (Boldarine et al., 2019). 

Geranial, neral, citronellal and geraniolursolic acid, oleanolic acid, caffeic acid, chlorogenic acid, quercetin, rhamnocitrin, and luteolin are the main ingredients of the MOE (Petrisor et al., 2022), which have shown antidepressant properties in animal models by increasing BDNF-mediated signaling (Dobrek et al., 2023; Takeda et al., 2006; Liu et al, 2017b; Hwang et al., 2016; Sharma et al., 2020). Rosmarinic acid is a polyphenol compound with antidepressant-like effects through modulation of mitogen-activated protein kinase phosphatase-1 and BDNF (Kondo et al., 2019). Other ingredients of MOE produced the beneficial effects of MOE such as central anti-oxidative and anti-apoptotic properties (Ghazizadeh et al., 2020). The results show that HFD caused a significant reduction of peripheral BDNF level, while in the treatment with MOE, and increase of serum BDNF levels suggesting that the beneficial effect of MOE might be ascribed to its modulatory effect on BDNF expression (Wang et al., 2010a; Abdel-Maksoud, 2017; Jahanban-Esfahlan et al., 2017).

Considering these results, it seems that the improving effects of MOE on HFD-induced biochemical and behavioral complications are due to its BDNF-dependent therapeutic effects. To our knowledge, this study provided novel experimental evidence for MOD efficacy in treating HFD-induced neurobehavioral consequences.

In conclusion, we report that MOE has several benefit effects on HFD-induced biochemical and behavioral adverse effects through modulation of serum BDNF levels in obese rats. Therefore, treatment with MOE therapy could provide an effective medication for obese patients with BDNF alterations.

**Table 1:** Serum lipid profiles of rats in each group.

	**Treatments**							
**mg/dl**	**Vehicle**	**HFD**	**ND+M50**	**ND+M100**	**ND+M150**	**HFD+M50**	**HFD+M100**	**HFD+M150**
Total cholesterol (TC)	73.4±3.43	103.2±4.01 ***	70±2.32	64.6±2.1	60.6±1.33	99.2±4.9	87.8±2.86 #	85±2.58 ##
Triglycerides (TG)	54.6±2.6	94.8±4.1 ***	51.2±3.85	45±3.38	40.6±2.9	93.4±2.1	81.6±2.6	74.6±2.5 ###
Low-density lipoprotein (LDL)	10.6 ±1.3	34.6 ± 2.2 ***	11.8± 1.1	11.6± 1	11.8± 1	32.2± 2.3	30±2.4	14.2± 1.1 ###
High-density lipoprotein (HDL)	30.2±2.4	14.6 ± 1.6 ***	31.2±2.33	32.6±2.54	34.2±2.44	35.4±2.32 ###	36±2.33 ###	39.4±3 ###

HFD: high fat diet; ND: normal diet; M50, M100 and M150: *Melissa officinalis *extract 50, 100 and 150 mg/kg/days for two weeks, respectively.
